# Effect of precipitating agent, N_2_ gas, extract volume and pH on the magnetic properties of magnetite nanoparticles by green synthesis from aqueous pomegranate peel extract

**DOI:** 10.3389/fchem.2024.1413077

**Published:** 2024-07-24

**Authors:** Marzieh Dehghani, Behnam Hajipour-Verdom, Parviz Abdolmaleki

**Affiliations:** Department of Biophysics, Faculty of Biological Sciences, Tarbiat Modares University, Tehran, Iran

**Keywords:** superparamagnetic nanoparticles, green synthesis, pomegranate peel extract, synthesis optimization, magnetic hyperthermia

## Abstract

Superparamagnetic nanoparticles (SPMNPs) have attracted considerable attention in biomedicine, particularly magnetic hyperthermia for cancer treatment. However, the development of efficient and eco-friendly methods for synthesizing SPMNPs remains a challenge. This study reports on a green synthesis approach for SPMNPs using pomegranate peel extract as a stabilizing agent. The effects of various synthesis parameters, including the type of precipitating agent (NH_3_ and NaOH), N_2_ gas, extract volume, and pH, were systematically investigated with regard to the size, morphology, and magnetic properties of the nanoparticles. The results showed that reducing the volume of the extract increased the saturation magnetization of the nanoparticles. N_2_ gas was found to be essential in preventing the oxidation of the nanoparticles. The type of precipitating agent also affected the size and magnetization of the nanoparticles, with NaOH leading to the synthesis of SPMNPs with higher magnetization (∼4 times) compared to NH_3_. Additionally, nanoparticles synthesized at pH 10 exhibited higher magnetization than those synthesized at pH 8 and 12. In conclusion, the optimized synthesis conditions significantly affected the magnetization and stability of SPMNPs. These nanoparticles are suitable for use in magnetic nanofluid hyperthermia applications.

## Introduction

Nanotechnology, which involves the study and manipulation of particles at the nanoscale, has revolutionized various fields, including biomedicine and industry (Khan et al., 2019; Lim et al., 2021). Among the diverse nanomaterials, magnetic nanoparticles, particularly superparamagnetic nanoparticles (SPMNPs), have gained significant attention due to their unique properties and wide-ranging applications (Shabatina et al., 2020; Chen et al., 2021). Their magnetic responsiveness allows for easy manipulation and separation, making them valuable for magnetic drug delivery, biosensing, and environmental remediation (Molaabasi et al., 2018; Shamsipur et al., 2019; Ashoori et al., 2023).

Superparamagnetic magnetite (Fe_3_O_4_) nanoparticles possess unique magnetic properties that make them highly suitable for various biomedical applications, particularly in magnetic hyperthermia (Kolen’ko et al., 2014). These properties include simple synthesis and characteristics, chemical stability, and biocompatibility (Xu and Sun, 2009). Moreover, the ability to generate heat under an alternating magnetic field (magnetic hyperthermia) enables their use in targeted cancer therapy (Ebrahimpour et al., 2021). Various factors contribute to heat generation, including the intensity and frequency of the applied magnetic field, nanoparticle concentration, size, shape, composition, and coating, as well as the viscosity of the medium and the synthesis method of the nanoparticles (Campos et al., 2015). Additionally, the synthesis method and conditions employed significantly influence the magnetic properties of these nanoparticles. Commonly employed techniques for magnetite nanoparticle synthesis include co-precipitation, sol-gel, microemulsion, thermal decomposition, and green synthesis (Campos et al., 2015; Rajan and Sahu, 2020).

Currently, magnetite nanoparticles can be synthesized by applying the green chemistry metrics. The goal of green chemistry is to design chemical products and processes that reduce or eliminate the use and production of hazardous substances. The green chemistry metrics include the following: 1- Preventing the creation of waste (prevention), 2- Maximizing the use of all reactants in creating the final product (atomic economy), 3- Minimizing toxicity for humans and the environment (less hazardous chemical syntheses), 4- Safer design of chemicals with proper performance (designing safer chemicals), 5- Using safer solvents and auxiliaries (safer solvents and auxiliaries), 6- Conducting reactions at ambient temperature and pressure (design for energy efficiency), 7- Use of renewable raw materials (use of renewable feedstocks), 8- Minimizing the use of unnecessary substances (blocking groups; protective groups) (reduce derivatives), 9- Using catalytic reagents instead of stoichiometric reagents (catalysis), 10- Synthesis of degradable products (design for degradation), 11- Controlling the reaction time and preventing the formation of dangerous substances (real-time analysis for pollution prevention), 12- Use of safer chemicals to minimize accidents (inherently safer chemistry for accident prevention) (Anastas et al., 2000).

Green synthesis methods for magnetite nanoparticles offer a promising approach over conventional synthesis techniques that utilize natural resources, such as plant extracts, as reducing and stabilizing agents (Dehghani and Ghadam, 2023). These methods provide environmentally friendly, non-toxic, cost-effective, and biocompatible nanoparticles with controlled properties, making them attractive for use in antimicrobial treatments, anti-cancer therapies, and other biomedical applications (Mustapha et al., 2022). Phytochemical compounds present in plant extracts play a crucial role in reducing metal ions to nanoparticles and stabilizing them. This process prevents agglomeration and ensures uniform size distribution (Venkataraman, 2022). A study by Sathishkumar et al. demonstrated the synthesis of magnetite nanoparticles using *Couroupita guianensis Aubl* fruit extract, highlighting their antimicrobial and anti-cancer properties. The nanoparticles exhibited a great antimicrobial effect against different human pathogens, indicating their potential as antimicrobial agents. Additionally, they showed a dose-dependent cytotoxic effect against human hepatocellular carcinoma cells (Sathishkumar et al., 2018). In the study conducted by Yousefi et al., magnetite nanoparticles were synthesized using *Garcinia mangostana* fruit peel extract, focusing on investigating their anti-cancer properties. They found that the magnetic nanofluids exhibited acceptable specific absorption rate (SAR) values and demonstrated thermosensitive performance during hyperthermia experiments (Yusefi et al., 2021).

The synthesis method and conditions play a significant role in determining the magnetic properties of nanoparticles (Hadadian et al., 2022). Several parameters can influence the magnetic properties of magnetite nanoparticles. These include temperature, pH, use of inert gases (such as N_2_ or Ar), precipitating agent, and the type and volume of the extract (Sathishkumar et al., 2018). The use of gas during synthesis can have a notable impact on the magnetization of nanoparticles, the formation of crystal structures, and the distribution of particle sizes. However, some studies provide examples of the different outcomes observed when N_2_ gas was not used in the synthesis of magnetite nanoparticles. Ba-Abbad et al. synthesized magnetite nanoparticles in nitrogen-free conditions, which had ferromagnetic properties (Ba-Abbad et al., 2022). In contrast, Etemadifar et al. and Dorniani et al. carried out similar syntheses without N_2_ gas and obtained nanoparticles with superparamagnetic properties (Dorniani et al., 2012; Etemadifar et al., 2018). These variations in magnetic properties could be attributed to the differences in the experimental conditions, such as the synthesis method, precursor materials, and reaction parameters. These parameters can significantly influence the size, shape, crystallinity, and surface properties of the nanoparticles, which in turn affect their magnetic behavior.

In this study, pomegranate peel (PP) extract was used as a coating and stabilizing agent for magnetite nanoparticles. PP extract is rich in bioactive compounds, with phenolic compounds being among the most important. The main phenolic compounds among them are tannins, flavonoids, phenolic acids, gallic acid, ellagic acid, catechin, and punicalagin (Fischer et al., 2011). Iranian PP extract is rich in phenolic compounds. For example, Ghasemi et al., (2023) reported, punicalagin, catechin, ellagic acid, and gallic acid account for 76.7%, 14.9%, 3.3%, and 3.1% of the eight monophenols recognized in pomegranate peel, respectively. Flavonoids in PP extract, with their various functional groups, play a crucial role in reducing metal ions and converting them into nanoparticles (Ramesh et al., 2015). Additionally, this study aims to optimize the synthesis conditions for obtaining superparamagnetic magnetite nanoparticles using a green synthesis approach. We investigated the role of N_2_ gas, type of precipitating agent, volume of the extract, and pH on the magnetic properties of the nanoparticles. The findings of this study advance the field of nanotechnology and pave the way for the development of new magnetic nanomaterials with improved performance and application in biomedical research, including magnetic nanofluid hyperthermia and targeted drug delivery.

## Materials and methods

### Chemical reagents

FeCl_2_.4H_2_O, FeCl_3_.6H_2_O and NaOH were purchased from Merck (Germany). NH_3_ and HCL were purchased from Sigma-Aldrich (Germany). Sweet pomegranate fruit was obtained from the orchards in Tehran province, Iran.

### Preparation of pomegranate peel extract

**FIGURE 1:**
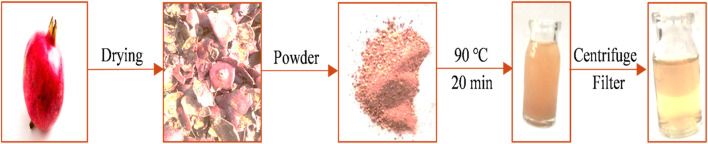
The preparation of pomegranate peel extract.

Fresh pomegranates were collected, de-seeded, and subsequently, the peel was carefully washed and cut into uniform pieces. The pomegranate peel (PP) dried for 2 weeks in the shade at 25°C to remove moisture and preserve its bioactive compounds. The dried PP was then ground into a fine powder using a home mill. To prepare the extract, 200 mg of the dry PP powder was added to 20 mL of distilled water in a heat-resistant container. The mixture was heated to 100°C to facilitate the extraction of bioactive compounds from the PP and then transferred to a water bath set at 90°C for 20 min. After cooling to room temperature, the extract was centrifuged at 4,000 rpm for 20 min. The supernatant was carefully collected and passed through a 0.2 µm filter. The final PP extract was stored in a refrigerator at 4°C to maintain its potency and prevent degradation (Figure 1).

### Synthesis and optimization of magnetite nanoparticles

**FIGURE 2:**
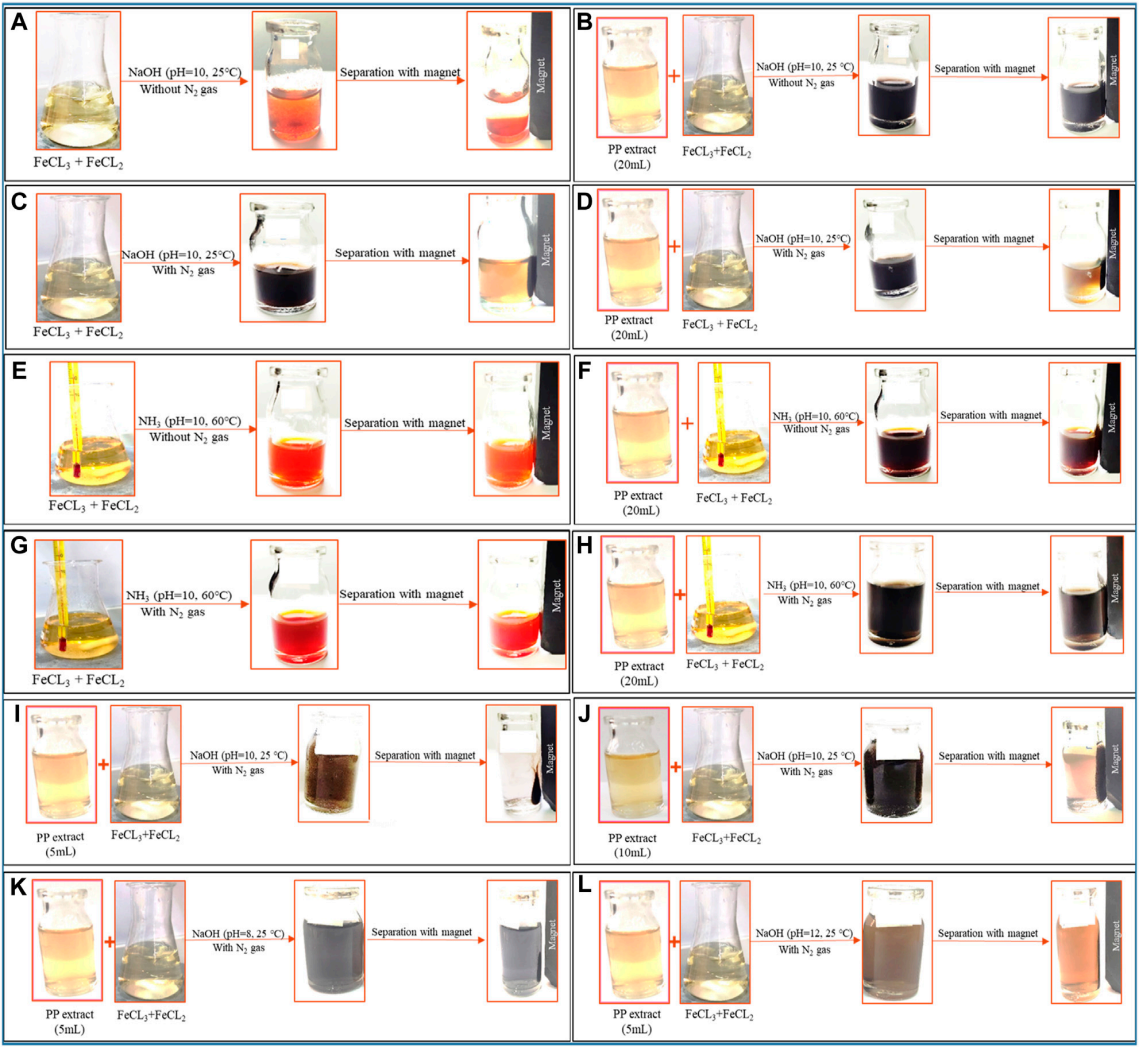
The synthesis of superparamagnetic magnetite nanoparticles was optimized through various conditions. Two different options for adjusting the pH were utilized: NaOH at room temperature and NH_3_ at 60°C under different conditions of **(A, E)** control (without Pomegranate peel (PP) extract and N_2_ gas), **(B, F)** with 20 mL PP extract (without N_2_ gas), **(C, G)** with N_2_ (without PP extract) and **(D, H)** with 20 mL PP extract/N_2_ at pH 10, respectively. The volume of PP extract was conducted using **(I)** 5 mL and **(J)** 10 mL in presence of NaOH and N_2_ gas at pH 10. The pH levels were optimized with 5 mL PP extract/N_2_ at different **(K)** pH 8 and **(L)** pH 12 in presence of NaOH.

To achieve superparamagnetic magnetite nanoparticles, the synthesis conditions were meticulously optimized. Initially, FeCL_2_ (1 mM) was stirred for 5 min, with the option of utilizing N_2_ gas. Subsequently, FeCL_3_ (2 mM) was added to the solution, and stirring continued for an additional 20 min. During the nucleation phase, the pH was adjusted to 10 using either NaOH at room temperature or NH_3_ at 60°C. Following the pH adjustment, the solution was stirred for an additional 20 min of stirring. The reaction then proceeded with or without the addition of 20 mL of PP extract (10 mg/mL) for 20 min. Subsequently, the samples were centrifuged at 4,000 rpm for 20 min. The resulting precipitate was washed thrice with distilled water and dried at 50°C for 4 h. Eight groups of synthesized magnetite nanoparticles were subjected to the necessary analyses. The synthesis steps and conditions are shown in Figures 2A–H; Table 1. Subsequently, the group exhibiting the most desirable superparamagnetic properties was selected, and its synthesis conditions were further optimized. The volume of the extract was investigated, considering 5 mL and 10 mL. The optimization steps and conditions for different extract volumes are illustrated in Figures 2I, J; Table 2. Additionally, pH values of 8 and 12 were evaluated for further pH optimization using NaOH. The steps and conditions involved in the pH optimization process are presented in Figures 2K, L; Table 3. Finally, the optimized sample underwent further analysis.

**TABLE 1:** Different conditions for synthesis of superparamagnetic magnetite nanoparticles using pomegranate peel (PP) extract with two different participating agents (NaOH and NH_3_) in presence and absence of N_2_ gas.

Samples	Precipitating agent (pH 10)		Iron source	N_2_ gas	PP extract solution (20 mL)
	NaOH	NH_3_			
Control	✓		FeCL_2_+FeCL_3_ (1:2)		
With extract	✓		FeCL_2_+FeCL_3_ (1:2)		✓
With N_2_	✓		FeCL_2_+FeCL_3_ (1:2)	✓	
With extract/N_2_	✓		FeCL_2_+FeCL_3_ (1:2)	✓	✓
Control		✓	FeCL_2_+FeCL_3_ (1:2)		
With extract		✓	FeCL_2_+FeCL_3_ (1:2)		✓
With N_2_		✓	FeCL_2_+FeCL_3_ (1:2)	✓	
With extract/N_2_		✓	FeCL_2_+FeCL_3_ (1:2)	✓	✓

**TABLE 2:** Optimizing the volume of pomegranate peel (PP) extract for synthesis of superparamagnetic magnetite nanoparticles in presence of NaOH, N_2_ gas and different volume of 5 and 10 mL at pH 10.

Sample	Precipitating agent	Iron source	N_2_ gas	pH	Volume of PP extract (mL)
With extract/N_2_	NaOH	FeCL_2_+FeCL_3_ (1:2)	✓	10	5
With extract/N_2_	NaOH	FeCL_2_+FeCL_3_ (1:2)	✓	10	10

**TABLE 3:** Optimization the pH level for synthesis of superparamagnetic magnetite nanoparticles in presence of NaOH, N_2_ gas and 5 mL pomegranate peel (PP) extract at different pH 8, 10 and 12.

Sample	Precipitating agent	Iron source	N_2_ gas	pH	Volume of PP extract (mL)
With extract/N_2_	NaOH	FeCL_2_+FeCL_3_ (1:2)	✓	8	5
With extract/N_2_	NaOH	FeCL_2_+FeCL_3_ (1:2)	✓	10	5
With extract/N_2_	NaOH	FeCL_2_+FeCL_3_ (1:2)	✓	12	5

### Structural characterization of magnetite nanoparticles

The optical properties of magnetite nanoparticles were investigated using a spectrophotometer (PerkinElmer Inc., Waltham, MA, United States) in the wavelength range of 200–800 nm. The surface plasmon resonance (SPR) band and UV/Vis absorption were assessed to gain insights into the optical behavior of the nanoparticles. Dynamic light scattering (DLS) and zeta potential measurements were performed using a Zetasizer Nano ZS (Malvern Instruments Ltd., United Kingdom) to determine the particle size distribution and surface charge, respectively. Magnetic properties were studied using a vibrating sample magnetometer (VSM) manufactured by Daghigh Kavir Engineering Company (Iran). Hysteresis curves were obtained at room temperature to calculate parameters such as coercivity (HC), saturation magnetization (MS), and magnetic susceptibility permeability. Fourier-transform infrared spectroscopy (FTIR) analysis was carried out using a TENSOR 27 spectrophotometer (Bruker Optik GmbH, Germany) in the wavelength range of 400 to 4,000 cm^–1^. This analysis provided information about the functional groups present on the surface of the nanoparticles (Kazemi-Ashtiyani et al., 2022). X-Ray diffraction (XRD) analysis was performed using an XRD Philips PW1730 (Netherlands) to investigate the crystal structure, grain size, interatomic distances, lattice parameters, and crystal defects of the nanoparticles. Field emission scanning electron microscope (FESEM) analysis was conducted using a FESEM ZEISS Sigma 300 (Germany) to obtain high-resolution images of the nanoparticles. Energy dispersive X-ray (EDAX) analysis was performed using the same FESEM instrument to identify the elemental composition and determine the weight or atomic percentage of each element present in the nanoparticles.

## Results and discussion

The use of green chemistry principles in the synthesis of nanomaterials has gained popularity in recent years (Mubayi et al., 2012). Moreover, Metal and metal oxide nanoparticles, including magnetite nanoparticles, have attracted significant attention due to their unique properties such as optical, catalytic, magnetic, and electrical characteristics (Sengupta and Sarkar, 2015; Zare-Moghadam et al., 2020). Magnetite nanoparticles have found diverse applications in catalysis, sensors, drug delivery, MRI, and magnetic hyperthermia (Laurent et al., 2008). Achieving superparamagnetic properties in magnetite nanoparticles is crucial for applications such as magnetic hyperthermia. Superparamagnetic magnetite nanoparticles possess a strong magnetic moment in the presence of an external magnetic field, but lose their magnetization once the field is removed. This property enables them to generate heat under an alternating magnetic field, which can be utilized for targeted cancer cell destruction in magnetic hyperthermia therapy. The challenge lies in synthesizing magnetite nanoparticles with superparamagnetic properties. However, by modifying the synthesis method and optimizing the relevant parameters, it is possible to obtain magnetite nanoparticles with the desired properties. This study aims to synthesize magnetite nanoparticles using green synthesis methods and optimize the synthesis conditions to achieve superparamagnetic properties suitable for magnetic hyperthermia. Eight different protocols were employed, involving variations in the precipitating agent (NaOH and NH_3_), the use of N_2_ gas, the volume of PP extract, and the pH of the reaction (Figure 2; Table 1).

### UV-Vis analysis confirms the synthesis of magnetite nanoparticles

**FIGURE 3:**
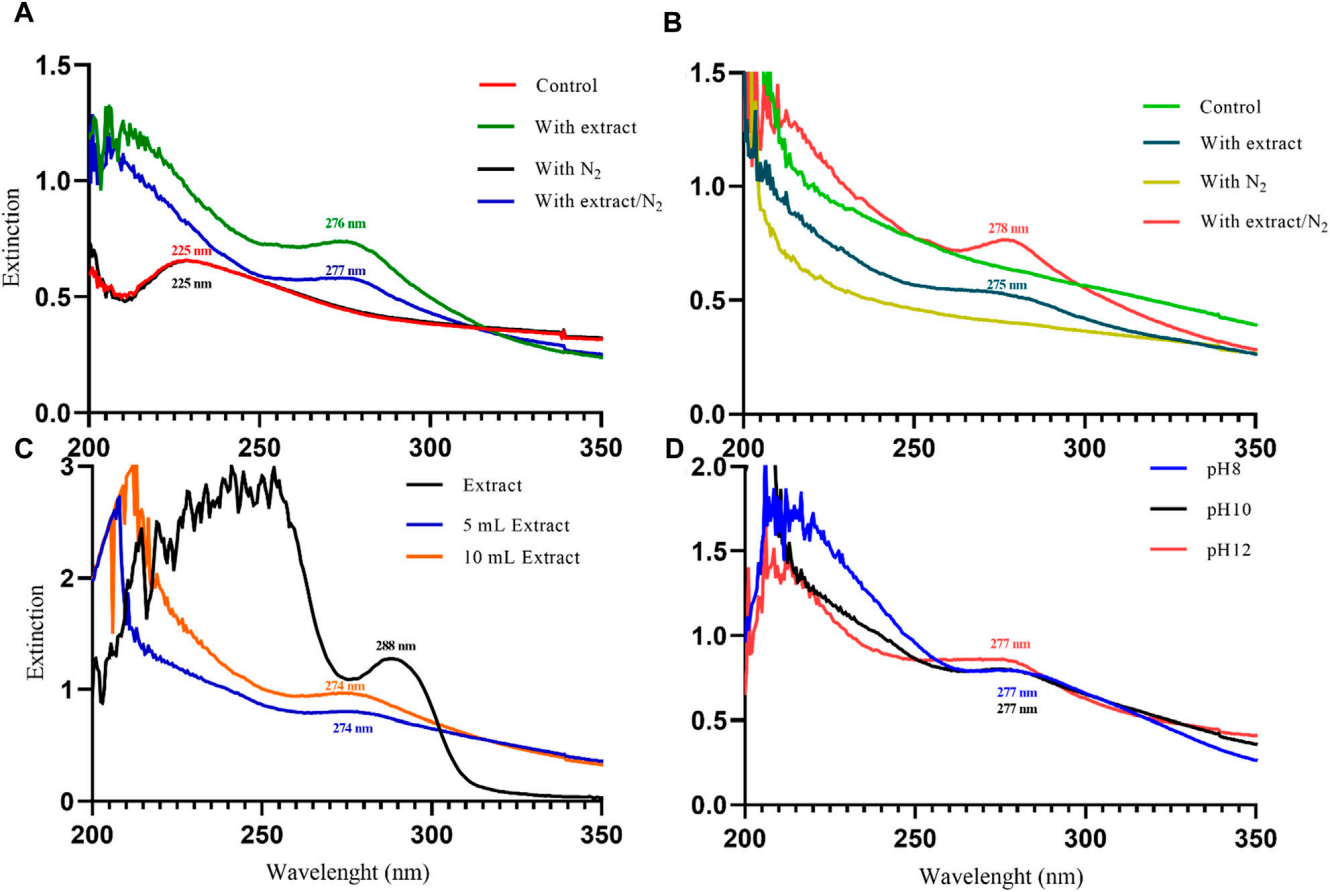
The UV-Visible spectroscopy analysis of magnetite nanoparticles was performed through various conditions. Different precipitating agents of **(A)** NaOH, **(B)** NH_3_ in presence and absence of 20 mL Pomegranate peel (PP) extract and N_2_ gas at pH 10. **(C)** Different volumes (5 and 10 mL) of PP extract in presence of NaOH and N_2_ gas at pH 10. **(D)** Different levels of pH in presence of NaOH, 5 mL PP extract and N_2_ gas.

UV-Vis spectrophotometry was performed on the suspension containing nanoparticles to confirm their synthesis. This analysis allows us to observe the surface plasmon resonance (SPR) band of the nanoparticles, which is a manifestation of the interaction between the conduction electrons of the nanoparticles and incident photons. The SPR band is sensitive to the size and shape of the nanoparticles, as well as the dielectric constant of the surrounding medium (Jana et al., 2016). In a study conducted by Akanda et al., (2023) in 2023, magnetite nanoparticles synthesized with *Tamarindus indica* leaf extract exhibited an SPR band at 280 nm. Similarly, Kiwumulo et al.,2022 synthesized magnetite nanoparticles using *Moringa oleifera* extract in 2022, which displayed a strong SPR band at 300 nm. In 2020, Azizi synthesized magnetite nanoparticles using tea extract, resulting in a wide SPR band ranging from 500 to 700 nm in the visible range (Azizi, 2020). In this study, magnetite nanoparticles synthesized using different protocols exhibited SPR bands in the UV range. Initially, when NaOH was used as the precipitating agent, the samples without extract (control and with N_2_) showed an SPR band at 225 nm. However, with the addition of 20 mL of PP extract, the SPR band showed a red shift to 276 nm (with extract) and 277 nm (with extract/N_2_). Indeed, with the increasing size of nanoparticles, the SPR band shows a red shift (Huang and El-Sayed, 2010). This indicates that the PP extract increased the size of the magnetite nanoparticles (Figure 3A).

In contrast, when NH_3_ was used as the precipitating agent, the samples without extract did not exhibit a clear SPR band. However, the samples containing the extract showed SPR bands at 275 nm (with extract) and 278 nm (with extract/N_2_) (Figure 3B). The observed red shift of the SPR band with increasing nanoparticle size is consistent with the literature. For particles larger than 100 nm, the SPR band broadening is also evident due to the dominant contributions from higher-order electron oscillations (Huang and El-Sayed, 2010). Overall, the UV-Vis analysis confirms the successful synthesis of magnetite nanoparticles and provides insights into the influence of synthesis conditions on their size and optical properties.

### Hydrodynamic diameter of magnetite nanoparticles

DLS analysis was performed to compare the hydrodynamic diameter of the synthesized nanoparticles. The hydrodynamic diameter refers to the size of the particles along with the “electrical double layer” formed by solvent molecules surrounding them. DLS relies on the Brownian motion of dispersed particles, where smaller particles exhibit rapid movements while larger particles display slower motion (Maguire et al., 2018). The results obtained from both UV-Vis spectroscopy and DLS analysis are in complete agreement. Samples containing PP extract demonstrated a larger hydrodynamic diameter, indicating that they are larger compared to the other groups. Indeed, the synthesis of nanoparticles includes three steps: nucleation, growth, and coating. If the aqueous extract of PP is added during the nucleation or growth stage. This behavior can be attributed to the role of PP extract in reducing iron ions, which leads to faster nucleation and faster growth of nanoparticles. But in this research, nucleation and growth were done, and then PP extract was added as a coating and stabilizing agent. Therefore, the larger size of the nanoparticles is due to the presence of functional groups of the extract on the surface of the magnetite nanoparticles (Skandalis et al., 2017).

**FIGURE 4:**
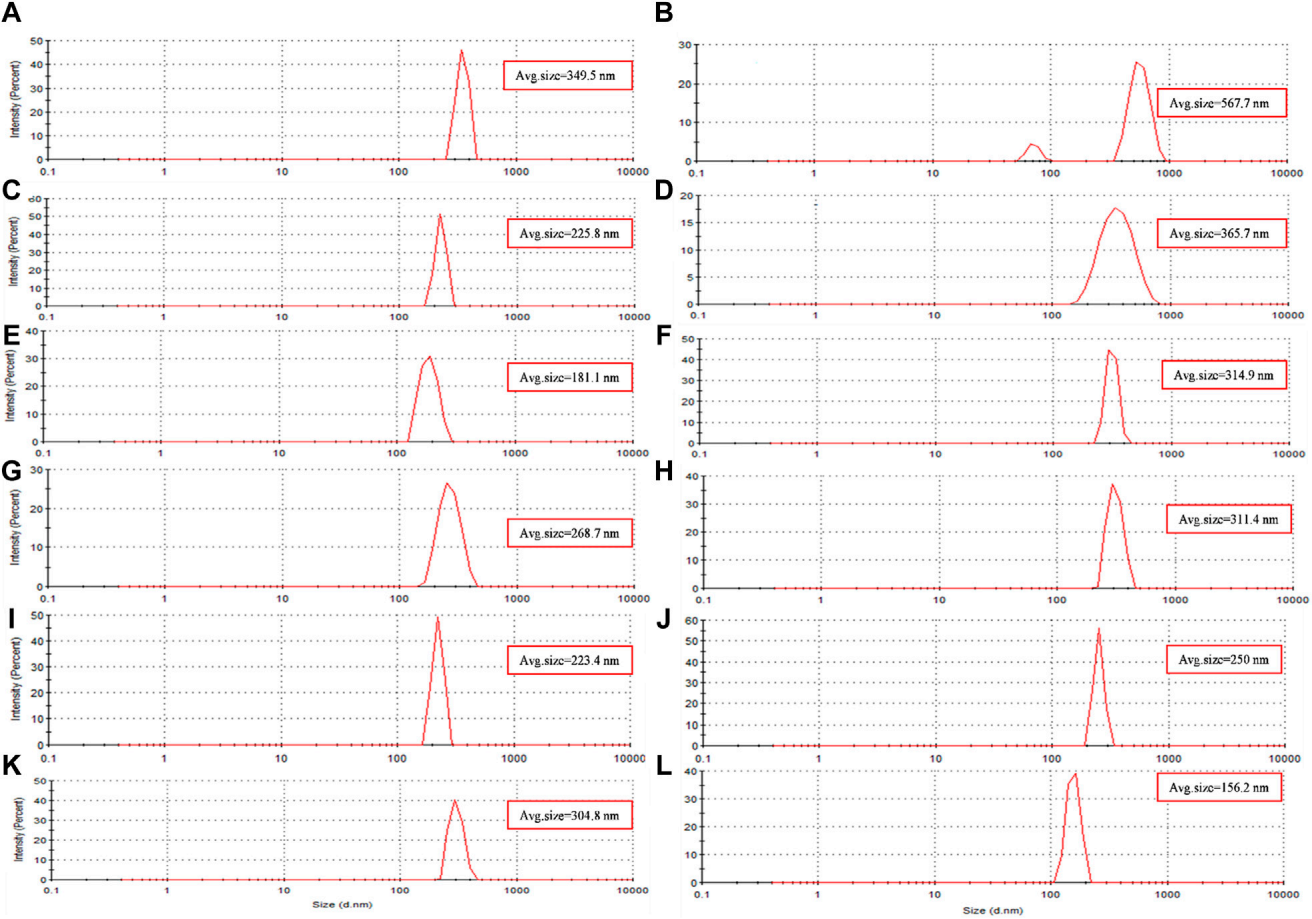
The size distribution diagram of synthesized magnetite nanoparticles was analyzed through various conditions. Two different precipitating agents of NaOH at room temperature and NH_3_ at 60°C under different conditions of **(A, E)** control (without Pomegranate peel (PP) extract and N_2_ gas), **(B, F)** with 20 mL PP extract (without N_2_ gas), **(C, G)** with N_2_ (without PP extract) and **(D, H)** with 20 mL PP extract/N_2_ at pH 10, respectively. Different volumes of PP extract at **(I)** 5 mL and **(J)** 10 mL in presence of NaOH and N_2_ gas at pH 10. pH levels at **(K)** pH 8 and **(L)** pH 12 in presence of NaOH and 5 mL PP extract/N_2_ gas.

The hydrodynamic diameter of the synthesized nanoparticles across the eight different protocols is summarized in Table 4. Additionally, Figures 4A–H presents the size distribution diagrams for the magnetite nanoparticles. For biomedical applications, the range of hydrodynamic diameter for nanoparticles should generally be less than 250 nm (Islam et al., 2020). All the synthesized magnetite nanoparticles met this criterion, indicating their potential suitability for biomedical applications.

**TABLE 4:** The hydrodynamic diameter of synthesized superparamagnetic magnetite nanoparticles in the presence and absence of N_2_ gas and 20 mL pomegranate peel extract using two participating agents of NaOH and NH_3_ at pH 10.

Sample	Precipitating agent (pH 10)		Hydrodynamic diameter (nm)
	NaOH	NH_3_	
Control	✓		349.5
With extract	✓		567.7
With N_2_	✓		225.8
With extract/N_2_	✓		365.7
Control		✓	188.1
With extract		✓	314.9
With N_2_		✓	268.7
With extract/N_2_		✓	311.4

### Surface charge of magnetite nanoparticles

Zeta potential analysis was performed to evaluate the surface charge of the synthesized nanoparticles. The zeta potential provides information about the surface charge of nanoparticles, which plays a crucial role in their long-term stability. The presence of surface charge on particles is the primary factor contributing to the stability of colloidal systems. When nanoparticles have similarly charged surfaces, they repel each other, preventing agglomeration or aggregation (DoymuŞ, 2007). Many factors such as the size of nanoparticles, the nature of the solution, ionic strength, concentration of dispersion, pH, buffer type, density, temperature, pressure, and formulation method such as the use of ultrasound affect the zeta potential (Kirby and Hasselbrink, 2004). The change in pH, Precipitating agent, temperature, and the volume of PP extract causes a change in the size of magnetite nanoparticles, which causes a change in the electric charge density on the surface of magnetite nanoparticles and as a result, increases the repulsive forces between them (Khairul et al., 2016).

**FIGURE 5:**
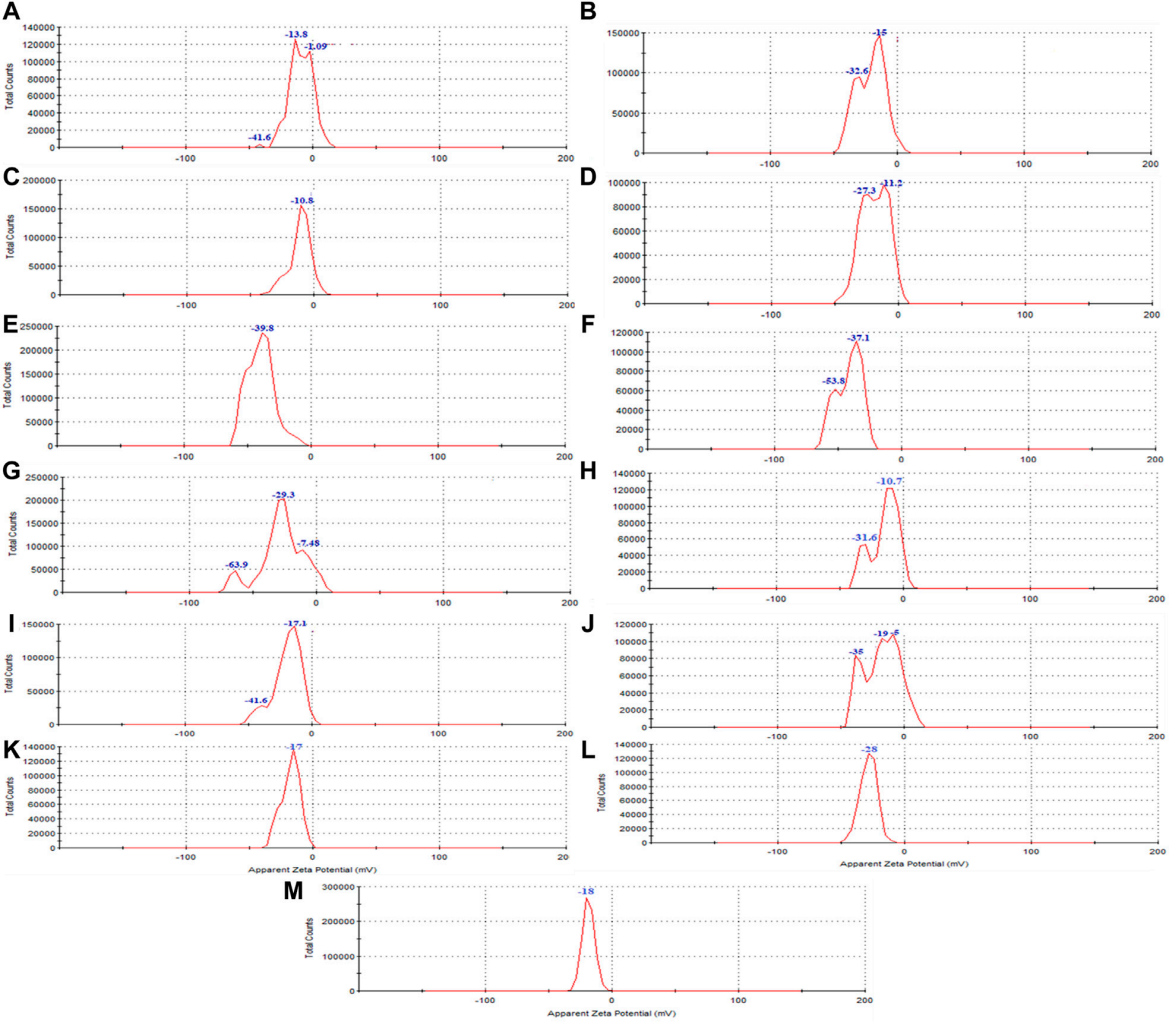
The zeta potential of synthesized magnetite nanoparticles was analyzed through various conditions. Two different precipitating agents of NaOH at room temperature and NH_3_ at 60°C under different conditions of **(A, E)** control (without Pomegranate peel (PP) extract and N_2_ gas), **(B, F)** with 20 mL PP extract (without N_2_ gas), **(C, G)** with N_2_ (without PP extract) and **(D, H)** with 20 mL PP extract/N_2_ at pH 10, respectively. Different volumes of PP extract at **(I)** 5 mL and **(J)** 10 mL in presence of NaOH and N_2_ gas at pH 10. Different pH levels at **(K)** pH 8 and **(L)** pH 12 in presence of NaOH and 5 mL PP extract/N_2_ gas. The zeta potential of the **(M)** PP extract solution was also examined in the double-distilled water.

The Zeta potential diagram and the charge distribution of the extract (Figure 5M) and magnetite nanoparticles are shown in (Figures 5A–H). The results indicate that the extract and all the samples of synthesized nanoparticles exhibited a negative zeta potential. A negative zeta potential suggests that the surfaces of the nanoparticles carry a net negative charge. This negative charge contributes to the electrostatic repulsion between particles, leading to their stability and preventing their aggregation or clumping.

### Magnetic properties of magnetite nanoparticles

**FIGURE 6:**
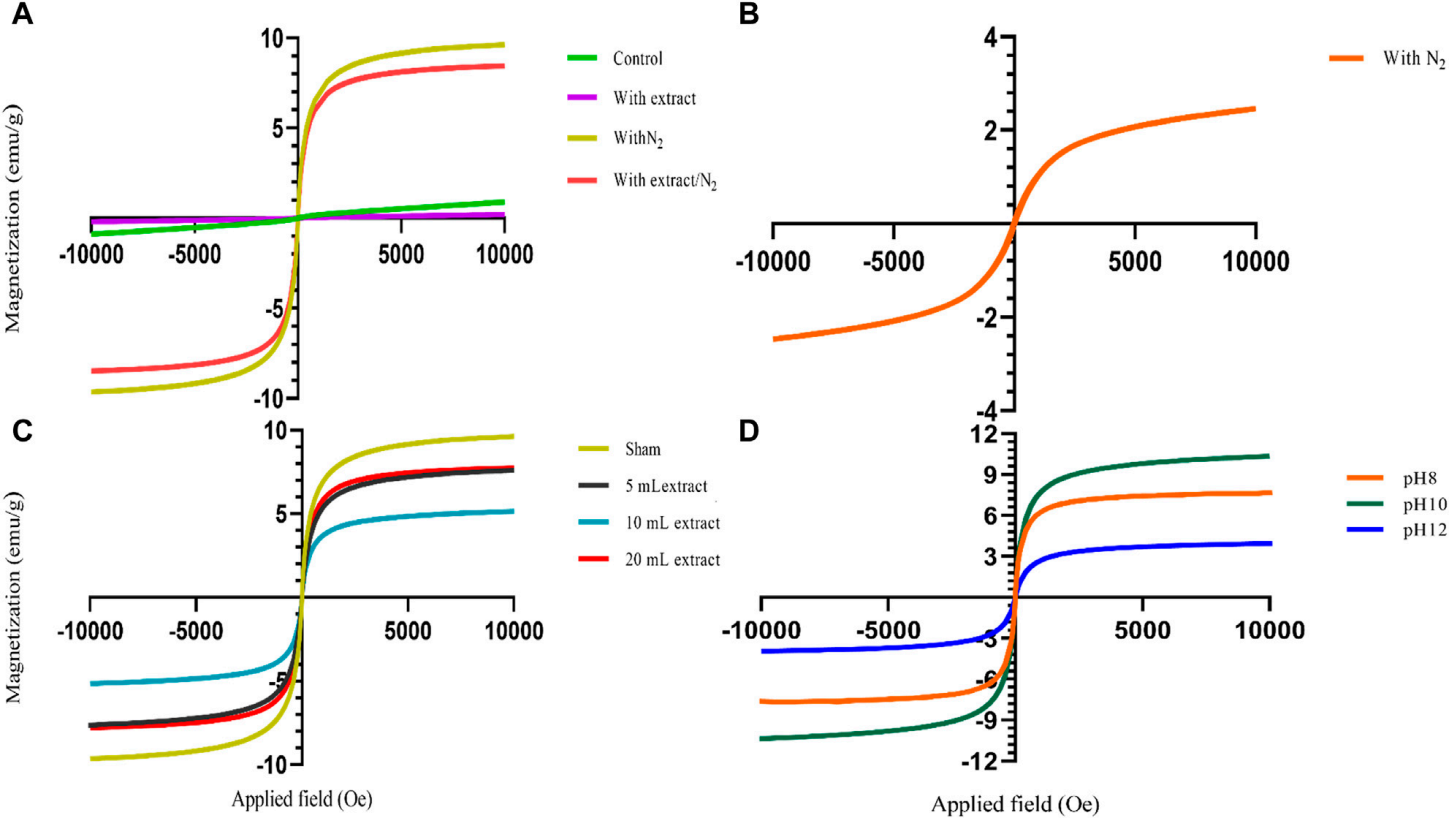
The vibrating sample magnetometry (VSM) diagram of synthesized magnetite nanoparticles was analyzed in various conditions. **(A)** Different groups of NaOH as a precipitating agent at room temperature, including control, with 20 mL Pomegranate peel (PP) extract, with N_2_ gas and with PP extract/N_2_ gas at pH 10. **(B)** The group only in presence of N_2_ gas with NH_3_ as a participating agent and without PP extract at pH 10. **(C)** Different volumes of PP extract (5, 10 and 20 mL) and sham group (without PP extract) in presence of NaOH and N_2_ gas at pH 10. **(D)** Different levels of pH at 8, 10, 12 in presence of NaOH, 5 mL PP extract and N_2_ gas.

The presence of different agents during the synthesis of nanoparticles can significantly impact their magnetic properties. As shown in Figures 2E–H, the use of NH_3_ as the precipitating agent resulted in nanoparticles that did not exhibit significant magnetic properties, while the use of NaOH, particularly in the presence of N_2_ gas, led to the acquisition of magnetic properties by the nanoparticles (Figures 2C, D). Because increasing the temperature affects the saturation magnetization of nanoparticles. Using NH_3_ as a precipitating agent at 60°C; decreased the saturation magnetization of magnetite nanoparticles. However, when NaOH was used as a precipitation agent at room temperature; the saturation magnetization of nanoparticles increased (Nkurikiyimfura et al., 2020). Presence of an external magnetic field can induce a strong magnetic moment in superparamagnetic nanoparticles, but they lose their magnetization when the field is removed, which is a desirable property for magnetic fluid hyperthermia applications. Moreover, VSM analysis provides information about the magnetic moment, saturation magnetization, and coercivity of the nanoparticles. The VSM analysis revealed that the nanoparticles synthesized under N_2_ gas exhibited superparamagnetic properties. On the other hand, the samples that were not synthesized under N_2_ gas demonstrated diamagnetic properties and were deemed unsuitable for magnetic fluid hyperthermia (Figures 6A, B). The formation of a non-magnetic layer such as extract on the surface of the nanoparticles decreased the magnetization of the nanoparticles, kwon as a dead magnetic layer (Elizondo-Villarreal et al., 2022). The saturation magnetization for the particles in presence of N_2_ gas was 10 emu/g, while in presence of extract/N_2_ gas was 7 emu/g. The results provide insights that the magnetic properties of magnetite nanoparticles are strongly influenced by their size and the presence of different agents during synthesis. The use of VSM and other characterization techniques is vital for understanding the behavior of these nanoparticles in different environments and their potential applications.

### Functional groups of extract and magnetite nanoparticles

**FIGURE 7:**
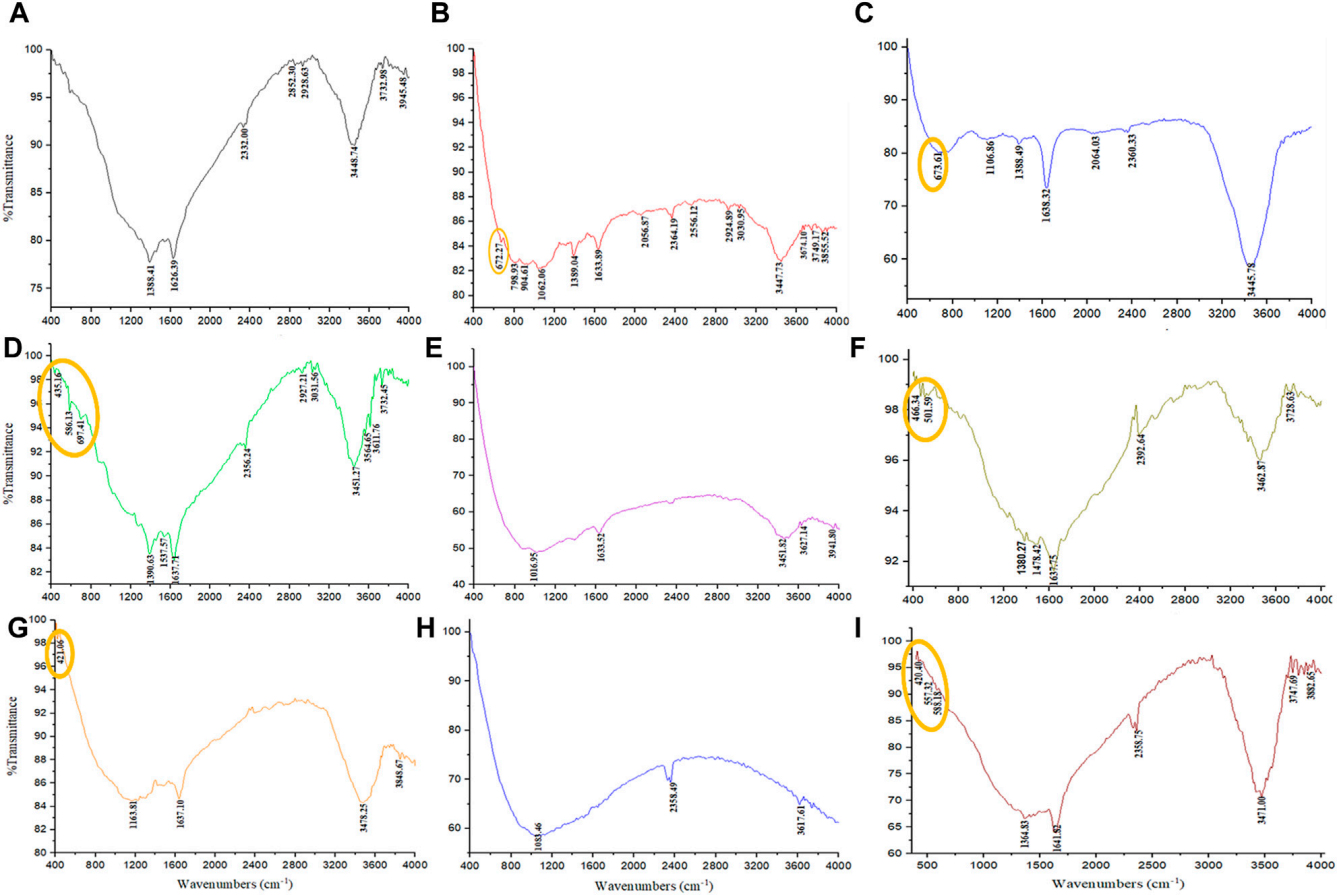
The FTIR spectrum were analyzed through various conditions. **(A)** PP extract solution (in the double-distilled water) and different groups of synthesized magnetite nanoparticles using NaOH as a precipitating agent at room temperature, including **(B)** control (without Pomegranate peel (PP) extract and N_2_ gas), **(C)** with 20 mL PP extract (without N_2_ gas), **(D)** with N_2_ gas (without PP extract), and **(E)** with 20 mL PP extract/N_2_ gas at pH 10, respectively. Different volumes of PP extract at **(F)** 5 mL and **(G)** 10 mL in presence of NaOH and N_2_ gas at pH 10. Different pH levels at **(H)** pH 8 and **(I)** pH 12 in presence of NaOH and 5 mL PP extract/N_2_ gas.

FTIR analysis was performed to identify the functional groups present in the PP extract and the magnetite nanoparticles. FTIR spectroscopy provides information about the chemical bonding and molecular structure of materials. The FTIR spectra of the PP extract and nanoparticles are shown in Figures 7A–E. The main peaks of the FTIR spectra are summarized in Table 5. The FTIR spectrum of the extract shows peaks at 3,945.48, 3,732.98, 3,448.74 cm^−1^ (O-H stretching), 2,928.63 and 2,852.30 cm^−1^ (C-H stretching), 1,626.39 cm^−1^ (C=O stretching), 1,388.41 cm^−1^ (C-H bending), and 2,332.00 cm^−1^ (C-O bending). These peaks indicate the presence of various functional groups, including hydroxyl, carbonyl, and alkene groups. For the magnetite nanoparticles synthesized with NaOH, the FTIR spectrum reveals a peak at 672.27 cm^−1^ (Fe-O stretching). However, when nanoparticles synthesized solely with NaOH and N_2_ gas, peaks are observed at 435.16 cm^−1^ (Fe-O stretching), 586.13 cm^−1^ (Fe-O stretching), and 697.41 cm^−1^ (Fe-O stretching). These peaks confirm the presence of iron oxide (magnetite) in the nanoparticles. The FTIR spectrum of the magnetite nanoparticles synthesized with NaOH and PP extract shows a peak at 673.61 cm^−1^ (Fe-O stretching), slightly shifted compared to the peak observed for the nanoparticles synthesized without extract. This shift may be due to the interaction between the extract and the magnetite nanoparticles. Notably, in the FTIR spectrum of the magnetite nanoparticles synthesized with NaOH, PP extract, and N_2_ gas does not show any Fe-O peaks.

**TABLE 5:** The FTIR spectrum were analyzed the pomegranate peel (PP) extract solution (in the double-distilled water) and different groups of synthesized superparamagnetic magnetite nanoparticles using NaOH as a precipitating agent, including control, with 20 mL PP extract, with N_2_ gas, and with 20 mL PP extract/N_2_ gas at pH 10.

Samples	Wave number (cm^−1^)	Band assignment	References
PP extract	3,945.48	O–H stretching	Kumar et al. (2014)
	3,732.98	O–H stretching	Kumar et al. (2014)
	3,448.74	O–H stretching	Kumar et al. (2014)
	2,928.63	C-H stretching	Kumar et al. (2014)
	2,852.30	C-H stretching	Kumar et al. (2014)
	2,332.00	C-O bending	Mishra et al. (2014)
	1,626.39	C=O stretching	Akshay et al. (2020)
	1,388.41	C-H bending	Trivedi et al. (2015)
Control	3,855.52	O-H stretching	Kumar et al. (2014)
	3,749.17	O-H stretching	Kumar et al. (2014)
	3,674.10	O-H stretching	Kumar et al. (2014)
	3,447.73	O-H stretching	Kumar et al. (2014)
	3,030.95	C-H stretching	Matrajt et al. (2004)
	2,924.89	C-H stretching	Kumar et al. (2014)
	2,556.12	S-H bending	Hakami et al. (2012)
	2,364.19	O=C=O stretching	Harris et al. (2002)
	2056.87	C=O stretching	Hakami et al. (2012)
	1,633.89	C=C stretching	Nandiyanto et al. (2016)
	1,389.04	C-H bending	Harris et al. (2002)
	1,062.06	C-O stretching	Buazar et al. (2016)
	904.61	C=C bending	Harris et al. (2002)
	798.93	C=C bending	Harris et al. (2002)
	672.27	Fe–O bonding	Buazar et al. (2016), Akshay et al. (2020)
With PP extract	3,445.78	O-H stretching	Kumar et al. (2014)
	2,360.33	O=C=O stretching	Harris et al. (2002)
	2064.03	C=C=N stretching	Hakami et al. (2012)
	1,638.32	C=C stretching	Nandiyanto et al. (2016)
	1,388.49	C-H bending	Harris et al. (2002)
	1,106.86	C-N stretching	Akshay et al. (2020)
	673.61	Fe–O bonding	Akshay et al. (2020)
With N_2_	3,732.45	O-H stretching	Kumar et al. (2014)
	3,611.76	O-H stretching	Kumar et al. (2014)
	3,564.65	O-H stretching	Kumar et al. (2014)
	3,451.27	O-H stretching	Kumar et al. (2014)
	3,031.56	C-H stretching	Matrajt et al. (2004)
	2,927.21	C-H stretching	Kumar et al. (2014)
	2,356.24	O=C=O stretching	Harris et al. (2002)
	1,637.71	C=C stretching	Nandiyanto et al. (2016)
	1,537.57	N-O stretching	Harris et al. (2002)
	1,390.63	C-H bending	Harris et al. (2002)
	697.41	Fe–O bonding	Akshay et al. (2020)
	586.13	Fe–O bonding	Buazar et al. (2016)
	435.16	Fe–O bonding	Mazrouaa et al. (2019)
with PP extract/N_2_	3,941.80	O-H stretching	Kumar et al. (2014)
	3,627.14	O-H stretching	Kumar et al. (2014)
	3,451.82	O-H stretching	Kumar et al. (2014)
	1,633.52	C=O stretching	Akshay et al. (2020)
	1,016.95	C-N stretching	Buazar et al. (2016)

### Optimization of extract volume for magnetite nanoparticle synthesis

After performing a series of characterization analyses, including UV-Vis, DLS, zeta potential, VSM, and FTIR, it was determined that the extract/N_2_ condition was the best group for further optimization. To optimize the volume of PP extract used in the synthesis, two additional volumes 5 and 10 mL of extract were investigated (Table 2). The nanoparticles synthesized with these different extract volumes were characterized, and the results were used to determine the optimal extract volume for synthesizing superparamagnetic magnetite nanoparticles with the desired magnetic properties.

The results of UV-Vis and DLS analyses provide insights into the effect of PP extract volume on the size of the magnetite nanoparticles. Both the nanoparticles synthesized with 5 mL and 10 mL of extract exhibit a SPR band at 274 nm in the UV-Vis spectra. This SPR band is blue-shifted compared to the peak observed for the PP extract at 288 nm (Figure 3C). The blue shift indicates a decrease in the size of the nanoparticles. This observation is confirmed by the DLS analysis, where the hydrodynamic diameter of the nanoparticles synthesized with 5 mL of extract is 223 nm, while that of the nanoparticles synthesized with 10 mL of extract is 250 nm (Figures 4I, J). Thus, increasing the volume of extract leads to an increase in the size of the nanoparticles. These findings suggest that the volume of PP extract plays a crucial role in controlling the size of the synthesized magnetite nanoparticles.

The zeta potential analysis shows indicates that both magnetite nanoparticles synthesized with 5 mL and 10 mL of extract possess a negative zeta potential, indicating a negative surface charge and contributing to their stability in aqueous solutions (Figures 5I, J). The synthesized nanoparticles with both extract volumes also exhibit magnetic behavior, as they are attracted by magnets (Figures 2I, J). VSM analysis confirms that both samples display superparamagnetic properties, meaning they possess a strong magnetic moment in the presence of an external magnetic field but lose their magnetization when the field is removed. However, the nanoparticles synthesized with 5 mL of extract exhibit a higher magnetization compared to those synthesized with 10 mL of extract (Figure 6C). This difference in magnetization may be attributed to the smaller size of the nanoparticles synthesized with 5 mL of extract, as smaller nanoparticles have larger magnetic domains, leading to stronger interaction with the magnetic field.

The surface functional groups of the synthesized nanoparticles were characterized using FTER analysis. The FTIR spectra reveal the presence of a distinct Fe-O band (Figures 7F, G). The main peaks observed in the FTIR spectra are summarized in Table 6. Notably, the magnetite nanoparticles synthesized using 5 mL of extract volume exhibit Fe-O peaks at 466.34 and 501.59 cm^−1^, while those synthesized with 10 mL of extract volume display a Fe-O peak at 421.06 cm^−1^.

**TABLE 6:** The FTIR analysis of synthesized superparamagnetic magnetite nanoparticles in presence of N_2_ gas and different pomegranate peel (PP) extract volume 5 and 10 mL using NaOH as participating agents at pH 10.

PP extract volume	Wave number (cm^−1^)	Band assignment	References
5 mL	3,728.63	O-H stretching	Kumar et al. (2014)
	3,462.87	O-H stretching	Kumar et al. (2014)
	2,392.64	O=C=O stretching	Harris et al. (2002)
	1,637.75	C=C stretching	Nandiyanto et al. (2016)
	1,478.42	C-O bending	Harris et al. (2002)
	1,380.27	C-H bending	Harris et al. (2002)
	501.59	Fe–O bonding	Buazar et al. (2016)
	466.34	Fe–O bonding	Mazrouaa et al. (2019)
10 mL	3,848.67	O-H stretching	Kumar et al. (2014)
	3,478.25	O-H stretching	Kumar et al. (2014)
	1,637.10	C=C stretching	Nandiyanto et al. (2016)
	1,163.81	C-N stretching	Harris et al. (2002)
	421.06	Fe–O bonding	Mazrouaa et al. (2019)

### Optimization of pH levels for magnetite nanoparticle synthesis

In the subsequent step, the magnetite nanoparticles synthesized using 5 mL of extract were selected based on their higher saturation magnetization and the presence of the Fe-O band. The pH of the synthesis process was then optimized by synthesizing nanoparticles at pH 8 and pH 12 (Table 3) to explore their magnetic susceptibility properties. Indeed, pH affects solubility. For ionic compounds containing basic anions, solubility is inversely proportional to pH. Solubility increases as the pH of the solution decreases. If the pH is higher than 3.5 the ferric iron (Fe^3+^) will become insoluble and precipitate but ferrous iron (Fe^2+^) precipitates at pH 8 (Balintova and Petrilakova, 2011).

The results from UV-Vis analysis indicate that both the nanoparticles synthesized at pH 8 and pH 12 exhibit SPR peaks at 277 nm (Figure 3D). This suggests that the nanoparticles have similar optical properties regardless of the pH.

DLS analysis reveales that the hydrodynamic diameter of the nanoparticles synthesized at pH 12 is 156 nm, while that of the nanoparticles synthesized at pH 8 is 304.8 nm (Figure 4K, L). The larger hydrodynamic diameter at pH 8 indicates an increase in the size of the nanoparticles compared to those synthesized at pH 12.

Zeta potential analysis indicates that both the nanoparticles synthesized at pH 8 and pH 12 have a negative zeta potential. The nanoparticles synthesized at pH 8 have a zeta potential of −17.6 mV, while those synthesized at pH 12 have a zeta potential of −28 mV (Figures 5K, L). The negative zeta potential suggests that the nanoparticles have a negatively charged surface, contributing to their stability in aqueous solutions.

FTIR analysis was conducted to evaluate the functional groups present on the surface of these nanoparticles. Notably, the nanoparticles synthesized at pH 8 did not exhibit the Fe-O band, whereas the Fe-O band was observed in the nanoparticles synthesized at pH 12 (Figures 7H, I). The presence of the Fe-O band confirms the formation of magnetite nanoparticles (Table 7).

**TABLE 7:** The FTIR analysis of synthesized superparamagnetic magnetite nanoparticles in presence of N_2_ gas and 5 mL pomegranate peel extract using NaOH as participating agents at different pH 8 and 12.

pH levels	Wave number (cm^−1^)	Band assignment	References
pH 8	3,617.61	O-H stretching	Kumar et al. (2014)
	2,358.49	C-O band	Mishra et al. (2014)
	1,083.46	C-O stretching	Buazar et al. (2016)
pH 12	3,882.65	O-H stretching	Kumar et al. (2014)
	3,747.69	O-H stretching	Kumar et al. (2014)
	3,471.00	O-H stretching	Kumar et al. (2014)
	2,358.75	C-O banding	Mishra et al. (2014)
	1,641.82	C=O stretching	Akshay et al. (2020)
	1,364.83	C-H banding	Trivedi et al. (2015)
	588.18	Fe–O bonding	Buazar et al. (2016)
	557.32	Fe–O bonding	Buazar et al. (2016)
	420.40	Fe–O bonding	Mazrouaa et al. (2019)

VSM analysis indicates that all synthesized magnetite nanoparticles exhibit superparamagnetic behavior at pH 8, 10, and 12. However, the nanoparticles synthesized at pH 10 demonstrate a higher magnetization compared to the other samples. Because in the range of pH 9.7 to 10.6, pure magnetite nanoparticles are synthesized. pH values below 8.5 lead to the formation of side-products to magnetite, specifically goethite and maghemite (Andrade et al., 2010) (Figure 6D).

Based on these findings, the optimal conditions for synthesizing magnetite nanoparticles include using NaOH as the participating agent, a 5 mL extract volume, conducting the synthesis under N_2_ gas atmosphere, maintaining a pH of 10, and performing the reaction at room temperature. Under these conditions, the nanoparticles exhibit superparamagnetic properties and achieve a higher level of magnetization compared to other synthesis conditions. Identifying these optimal conditions is crucial as it enables researchers to determine the most favorable parameters for synthesizing magnetite nanoparticles with the desired magnetic properties. These SPMNPs with enhanced magnetization hold significant potential for various applications, including magnetic separation, biomedical diagnostics, and targeted drug delivery systems.

### More analysis of magnetite nanoparticles in the optimized synthesis conditions

**FIGURE 8:**
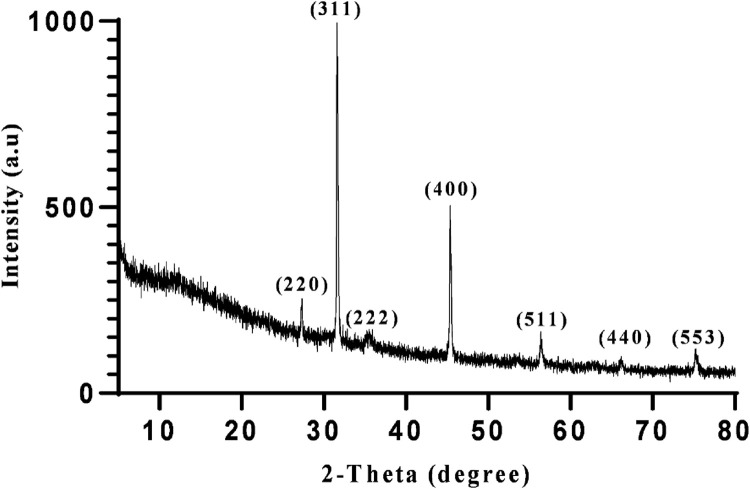
The XRD profile of synthesized superparamagnetic magnetite (Fe_3_O_4_) nanoparticles using NaOH with 5 mL Pomegranate peel extract in presence of N_2_ gas at pH 10 as optimum conditions.

In the following step, XRD, FESEM, and EDAX analysis were performed to fully characterize the optimized magnetite nanoparticles synthesized using 5 mL of extract at pH 10. XRD analysis was conducted to confirm the crystal structure and phase purity of the synthesized nanoparticles. As shown in Figure 8, the XRD patterns confirmed the synthesis of magnetite nanoparticles. The diffraction peaks observed in the XRD pattern correspond to specific crystallographic planes, such as 220, 311, 222, 400, 511, 440, and 533 (Silva et al., 2013). The presence of sharp peaks suggests a crystalline nature of the nanoparticles, while the presence of broad peaks indicates the presence of ultra-fine and small crystallite sizes (Medina-Zazueta et al., 2023).

**FIGURE 9:**
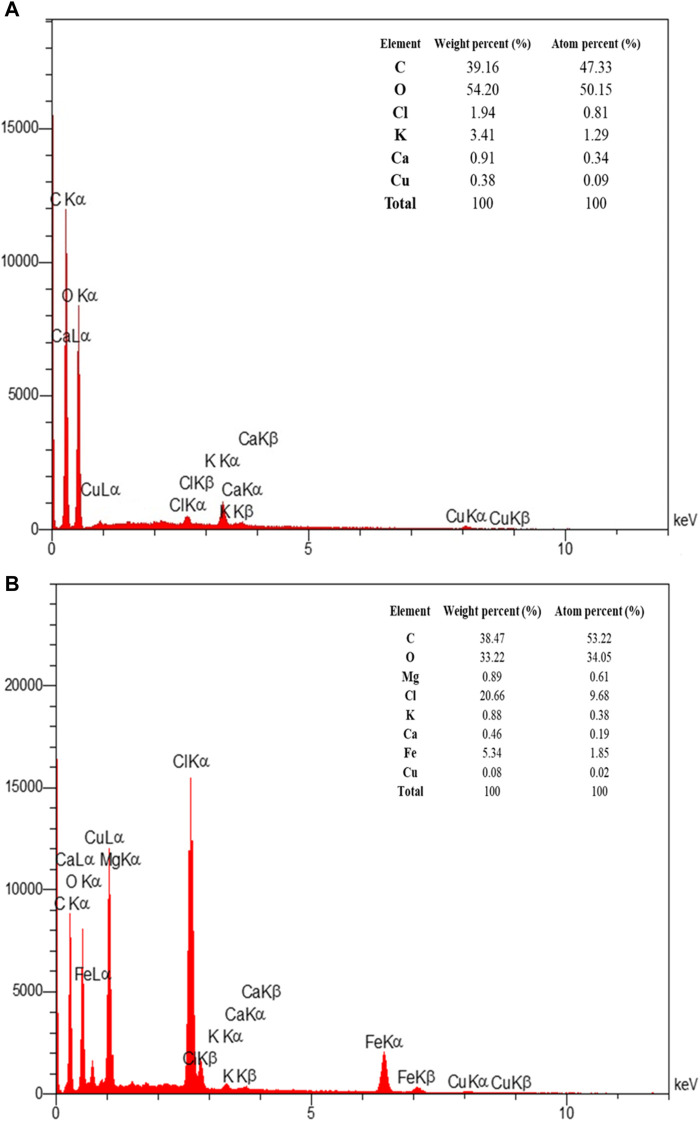
The EDAX spectra of **(A)** Pomegranate peel extract solution (in the double-distilled water) and **(B)** synthesized superparamagnetic magnetite (Fe_3_O_4_) nanoparticles using NaOH with 5 mL PP extract volume in presence of N_2_ gas at pH 10 as optimum conditions.

EDAX analysis was used to determine the elemental composition of both the extract and the optimized magnetite nanoparticles. Figure 9 displays the EDAX spectrum of the extract and magnetite nanoparticles. The analysis confirms that the magnetite nanoparticles synthesized under the optimized conditions contain compositions of iron and oxygen. This analysis provides quantitative information about the percentage of these elements present in the nanoparticles, verifying their elemental composition.

**FIGURE 10:**
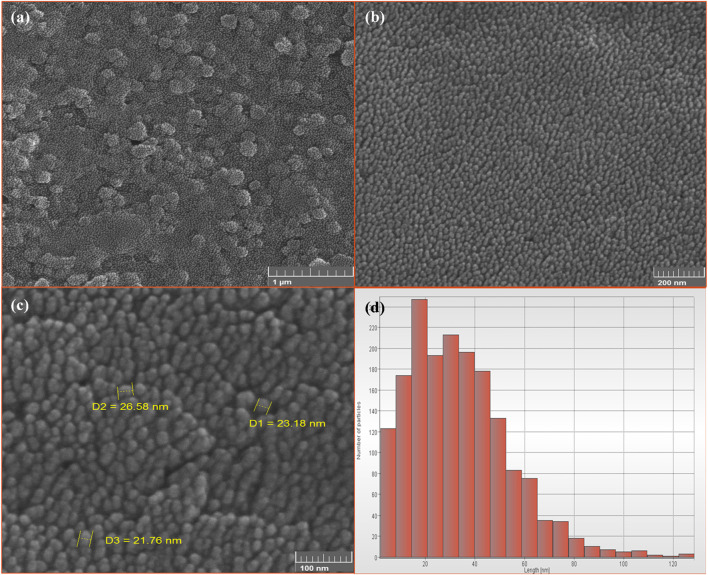
The FE-SEM images of synthesized superparamagnetic magnetite (Fe_3_O_4_) nanoparticles using NaOH with 5 mL PP extract volume in presence of N_2_ gas at pH 10 with different resolution. Scale bars: **(A)** 1 μm, **(B)** 200 nm and **(C)** 100 nm. **(D)** The size distribution of Fe_3_O_4_ nanoparticles.

FESEM analysis results indicate that the synthesized magnetite nanoparticles are monodisperse and exhibit a spherical shape. Figure 10 provides visual evidence of the nanoparticles at different resolutions, showcasing their size distribution. The average size of the synthesized nanoparticles is approximately 25 nm, falling within the recommended range of 10–100 nm for nanoparticles used in magnetic nanofluid hyperthermia (Chang et al., 2018). Nanoparticles smaller than 10 nm are generally ineffective in heat generation. The monodispersity and superparamagnetic properties of the synthesized magnetite nanoparticles make them suitable for generating uniform and effective heat in magnetic nanofluid hyperthermia applications (Nguyen and Kim, 2016).

## Conclusion

The green synthesis of superparamagnetic magnetite nanoparticles using biological extracts has emerged as a highly attractive method, renowned for its safety, cost-effectiveness, and eco-friendliness. Various studies, including those utilizing different biological sources such as *Citrus sinensis* peel extract, *M. oleifera*, and *Eucalyptus globulus* extract, have demonstrated successful green synthesis (Andrade-Zavaleta et al., 2022; Kiwumulo et al., 2022; Eldeeb et al., 2023). In this study, we prioritized green chemistry metrics, employing the aqueous extract of PP as a stabilizer for magnetite nanoparticles. Notably, the synthesis occurred under ambient pressure, avoiding the generation of toxic waste, high temperatures, and hazardous solvents. Exploring the optimization of synthesis parameters, including the PP extract, N_2_ gas, precipitating agents, and pH variations, showed crucial. Our research underscores the promise of this green synthesis method for diverse applications, notably in magnetic nanofluid hyperthermia and targeted drug delivery systems. The synthesized nanoparticles exhibit desirable characteristics such as biocompatibility, stability, and suitable magnetic properties, positioning them favorably for biomedical applications.

Optimized conditions, such as the presence of N_2_ gas, NaOH as a precipitating agent, specific extract volumes, and pH levels, significantly influence nanoparticle magnetization. Notably, our findings highlight pH 10 as optimal for magnetite nanoparticle synthesis, ensuring both purity and stability. Furthermore, our study elucidates the impact of pH on nanoparticle solubility and precipitation, crucial considerations for synthesis optimization. The development of magnetic nanomaterials with enhanced performance, as facilitated by this study, holds immense promise for biomedicine. By addressing factors influencing magnetization and synthesizing nanoparticles under optimized conditions, we advance the field, paving the way for improved applications in magnetic hyperthermia and targeted drug delivery. In conclusion, the green synthesis of superparamagnetic magnetite nanoparticles using biological extracts presents a sustainable and effective approach with vast potential in biomedicine. Our study contributes valuable insights into synthesis optimization and nanoparticle characterization, driving innovation and progress in the development of magnetic nanomaterials for biomedical applications.

## Data Availability

The original contributions presented in the study are included in the article/Supplementary Material, further inquiries can be directed to the corresponding author.
